# Supplementary figures and images for: A genome-wide association analysis reveals a potential role for recombination in the evolution of antimicrobial resistance in Burkholderia multivorans
Source: PLoS Pathog. 2018 Dec 7;14(12):e1007453. doi: 10.1371/journal.ppat.1007453 (PMC6300292; doi:10.1371/journal.ppat.1007453)

**A**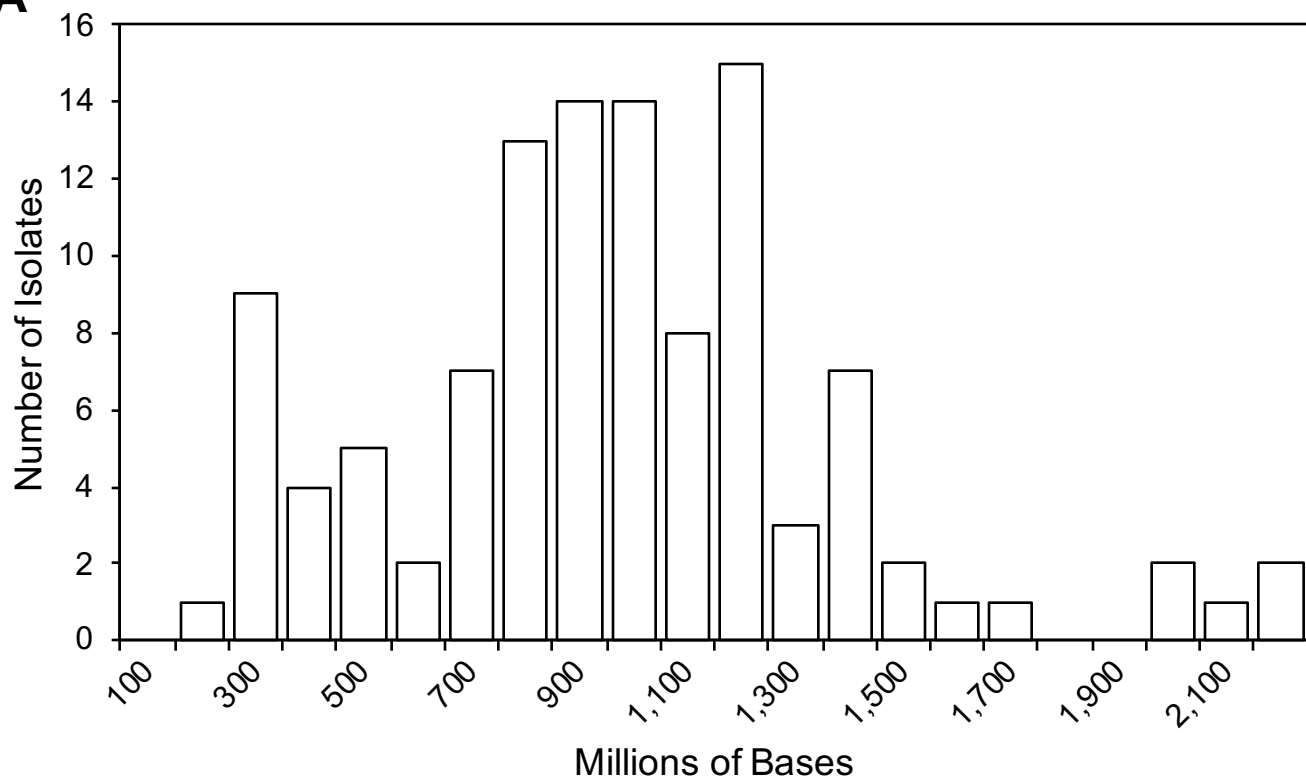**B**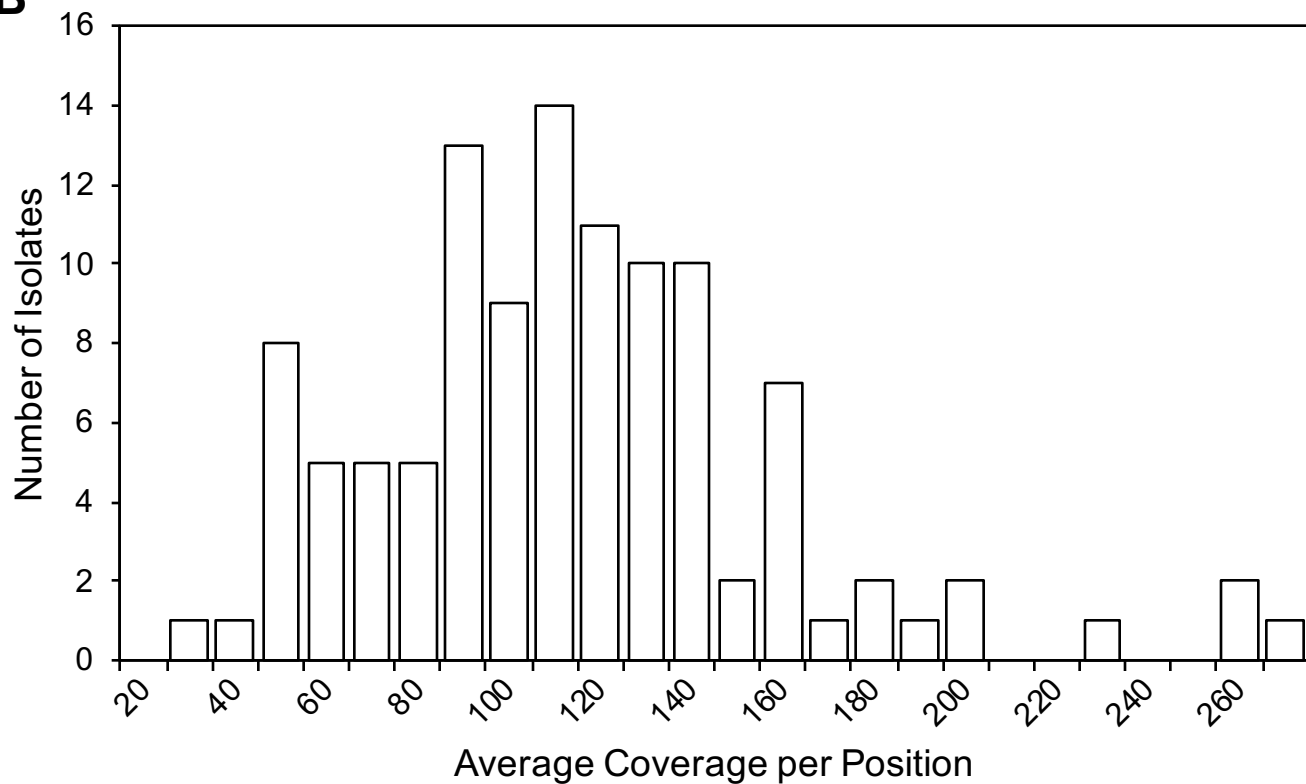

Supplement: S1 Fig — Whole genome sequencing of 111 isolates of B. multivorans in the Illumina platform. (A) Distribution of number of bases sequenced per isolate. (B) Distribution of median read depth per position. (PDF) [file ppat.1007453.s001.pdf]

**A**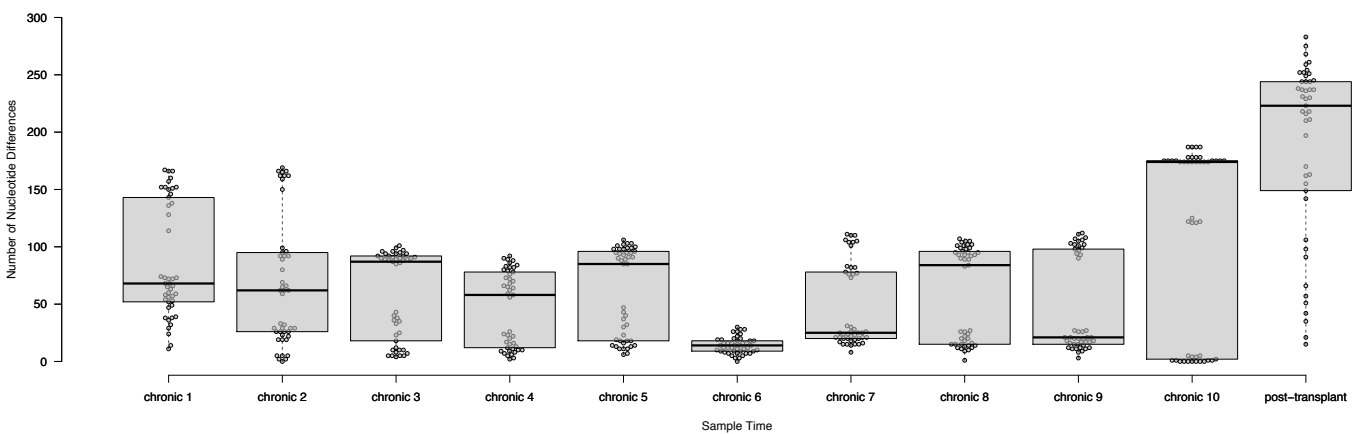**B**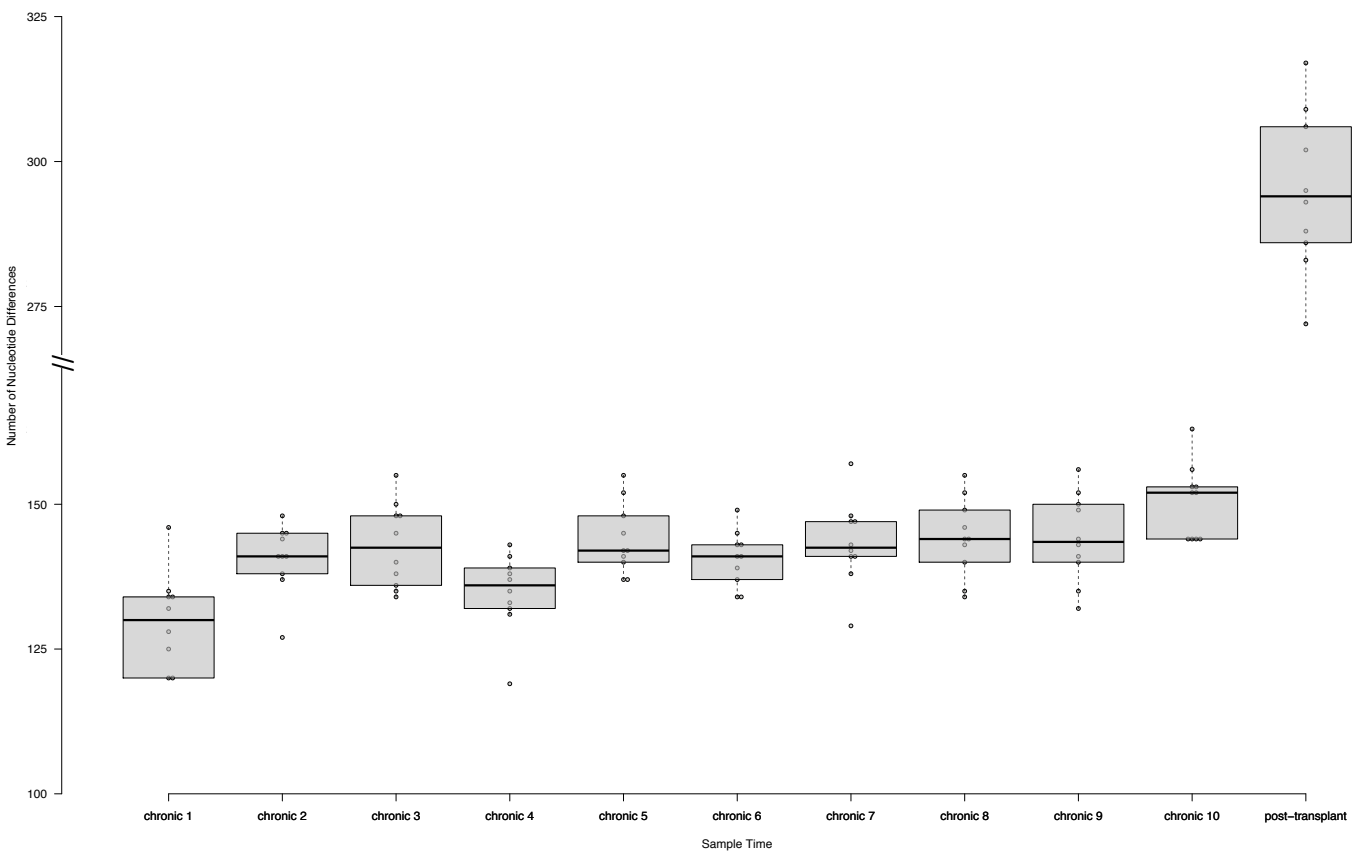

Supplement: S2 Fig — (A) Pairwise nucleotide differences between isolates collected from the same collection sample. Incident infection is not included since only one isolate was recovered from that time point. (B) Nucleotide differences between each isolate and the incident infection isolate. (PDF) [file ppat.1007453.s002.pdf]

A

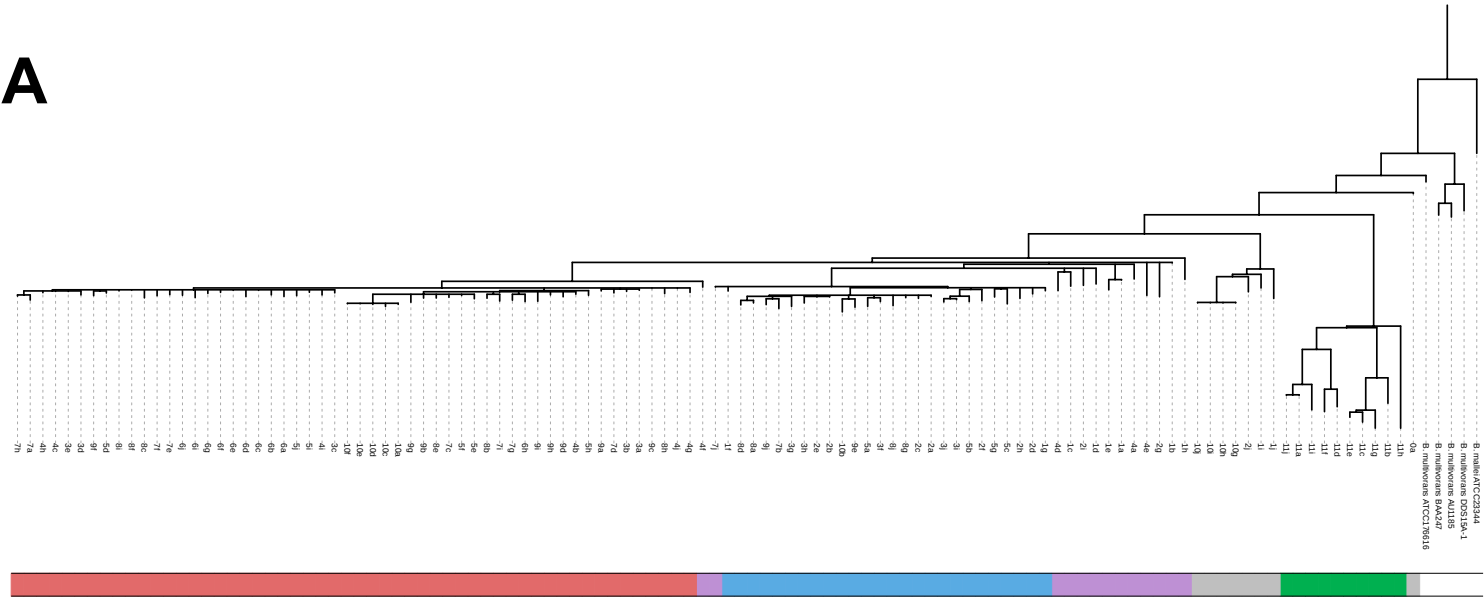

B

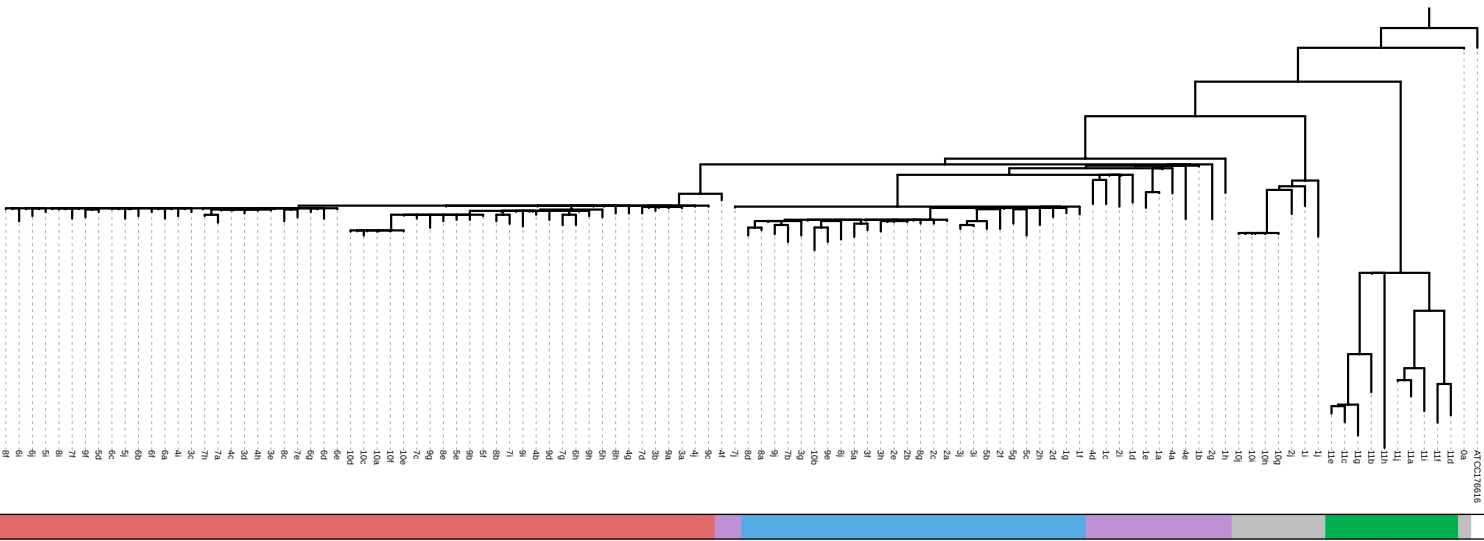

C

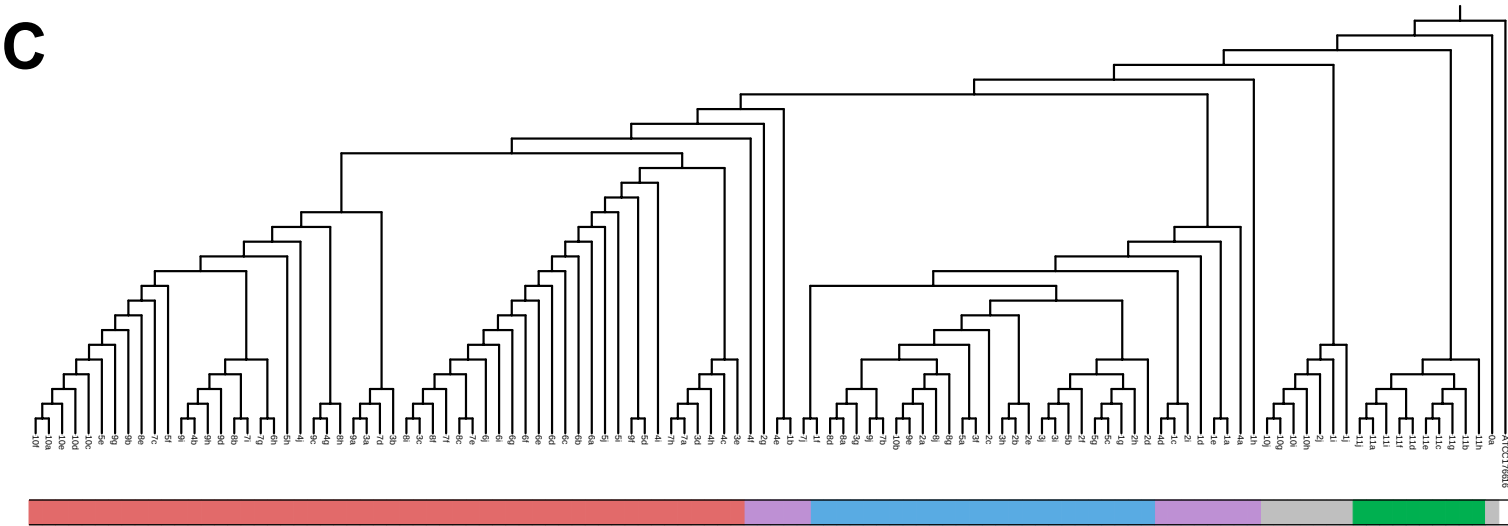

D

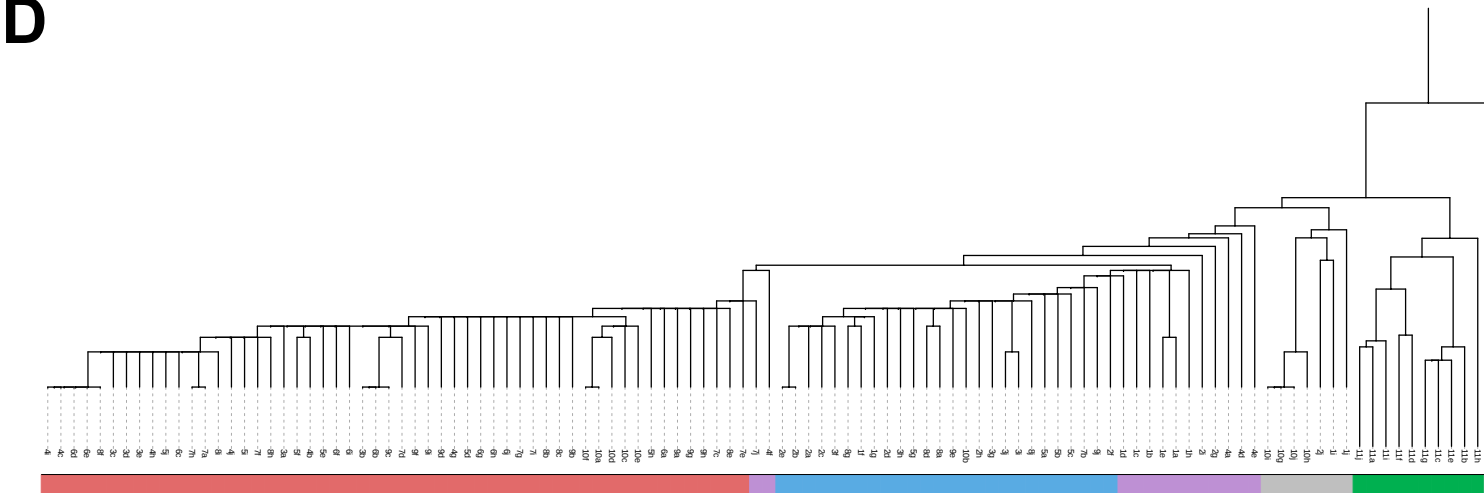

Supplement: S3 Fig — A) Maximum likelihood phylogeny including B. multivorans ATCC 17616, B. multivorans BAA247, B. multivorans DDS15A-1, and B. multivorans AU1185 from the Burkholderia Genome database [68]. This tree was estimated using the General Time Reversible (GTR) model in MEGA7 with 500 bootstrap iterations, and it was rooted with B. mallei ATCC 23344 as the outgroup [81]. B) Maximum likelihood phylogeny rooted using B. multivorans ATCC 17616 as the outgroup. This tree was estimated under the GTR model in MEGA7 using 500 bootstrap iterations [81]. C) Maximum parsimony phylogeny rooted with B. multivorans ATCC 17616 as the outgroup. This tree was estimated using MEGA7 and 500 bootstrap iterations [81]. D) Hierarchical clustering based on the presence and absence of insertions or deletions among the 111 isolates using Euclidian distances as implemented by the vegan package in R [93]. This dendrogram was rooted with the incident isolate as the outgroup. (PDF) [file ppat.1007453.s003.pdf]

Time scale: 0.01

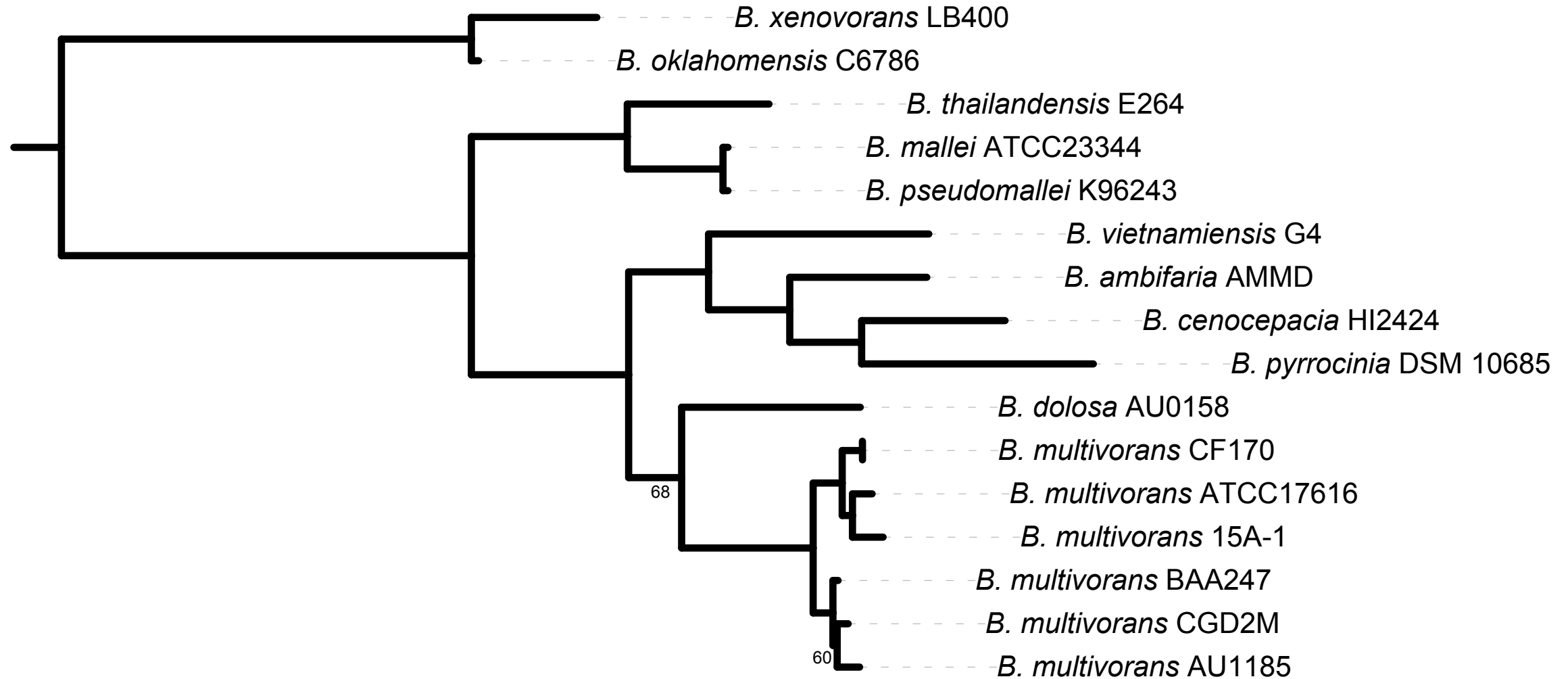

Supplement: S4 Fig — The sequences of seven housekeeping genes (atpD, gltB, gyrB, lepA, phaC, recA, and trpB) from B. xenovorans LB 400, B. oklahomensis C6786, B. thailandensis E264, B. mallei ATCC 23344, B. pseudomallei K96243, B. vietnamiensis G4, B. ambifaria AMMD, B. cenocepacia HI2424, B. pyrrocinia DSM 10685, B. dolosa AU 0158, B. multivorans ATCC 17616, B. multivorans 15A-1, B. multivorans BAA 247, B. multivorans CGD2M, and B. multivorans AU1185 were extracted as defined by pubMLST [24]. These sequences were aligned with MUSCLE (default parameters) [94], and the resulting alignment was used to recreate their phylogenetic relationships with a Maximum Likelihood approach (Bootstrap = 1,000). (PDF) [file ppat.1007453.s004.pdf]

0.02

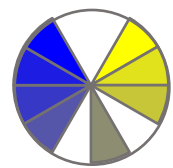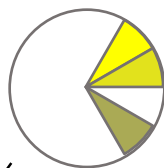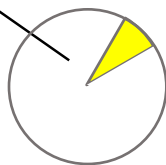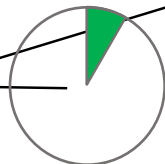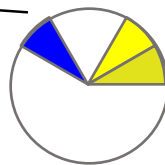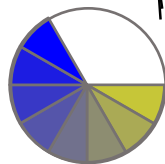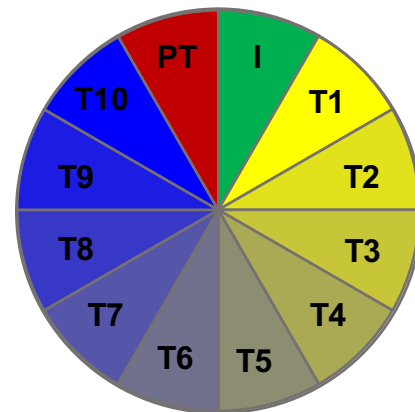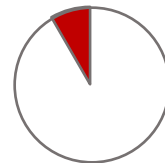

Supplement: S5 Fig — This network-based phylogeny was calculated in SplitsTree v. 4.14.4. Individual strain names at the tips of each branch have been replaced with pie charts indicating the distribution of dates during which the strains were sampled (indicated by the circular legend). (PDF) [file ppat.1007453.s005.pdf]

**A**

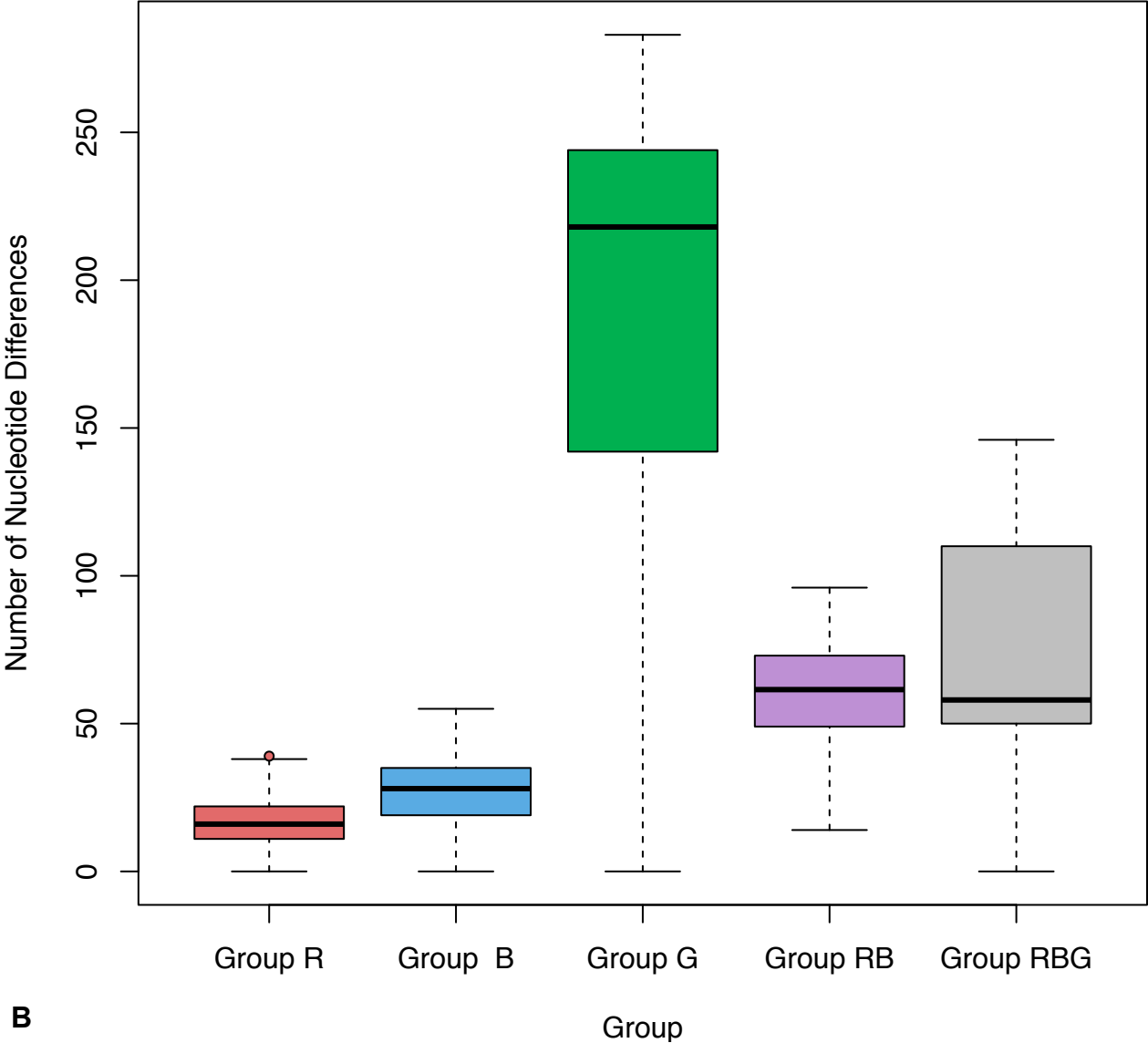

**B**

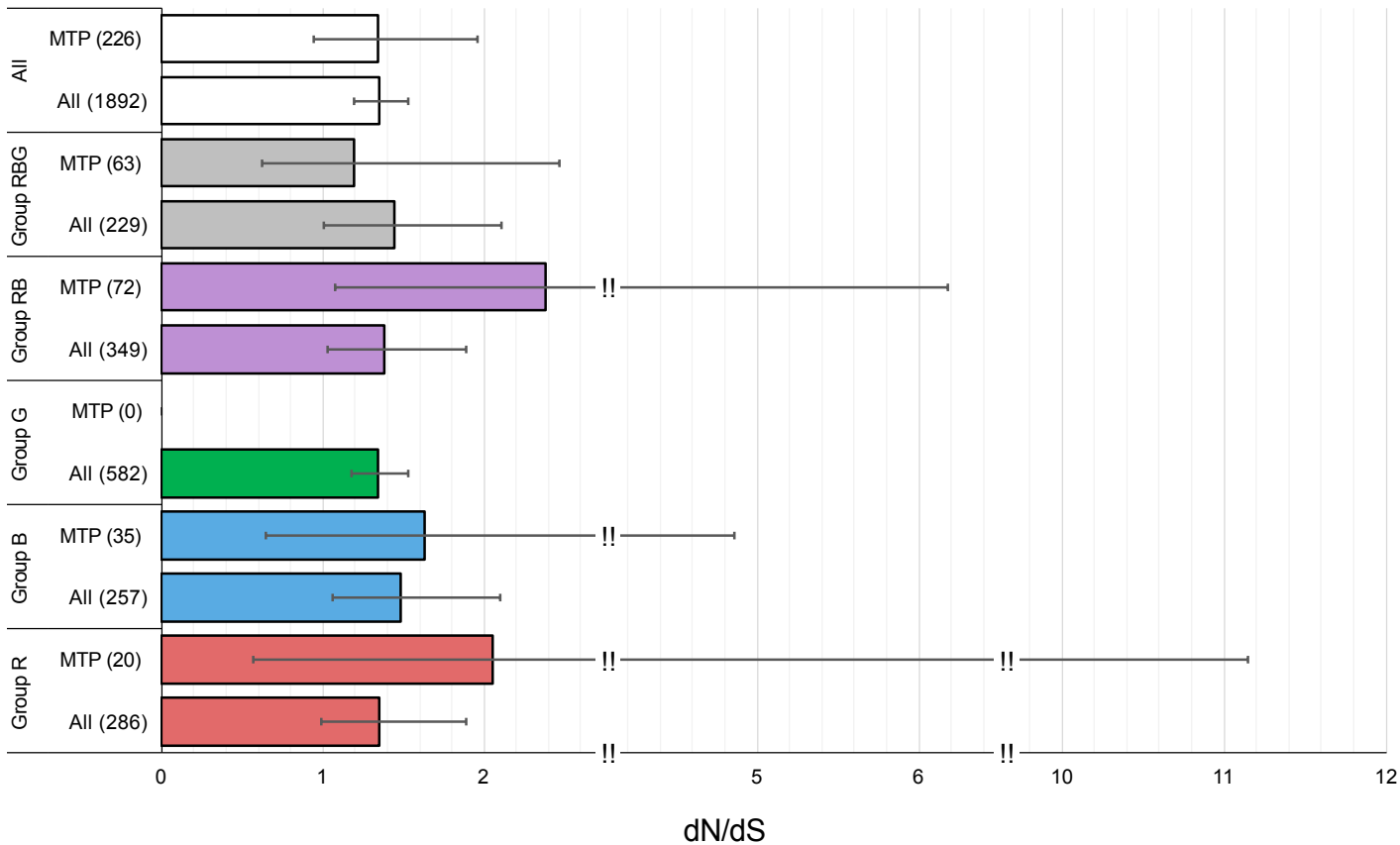

Supplement: S6 Fig — (A) Pairwise nucleotide differences between isolates from the same group based on ancestry. (B) dN/dS per group calculated including all SNPs and using only SNPs observed in multiple time points (MTP). dN/dS and the respective confidence intervals were calculated as described by Lieberman et al. [87]. (PDF) [file ppat.1007453.s006.pdf]

Tree scale: 0.01

A

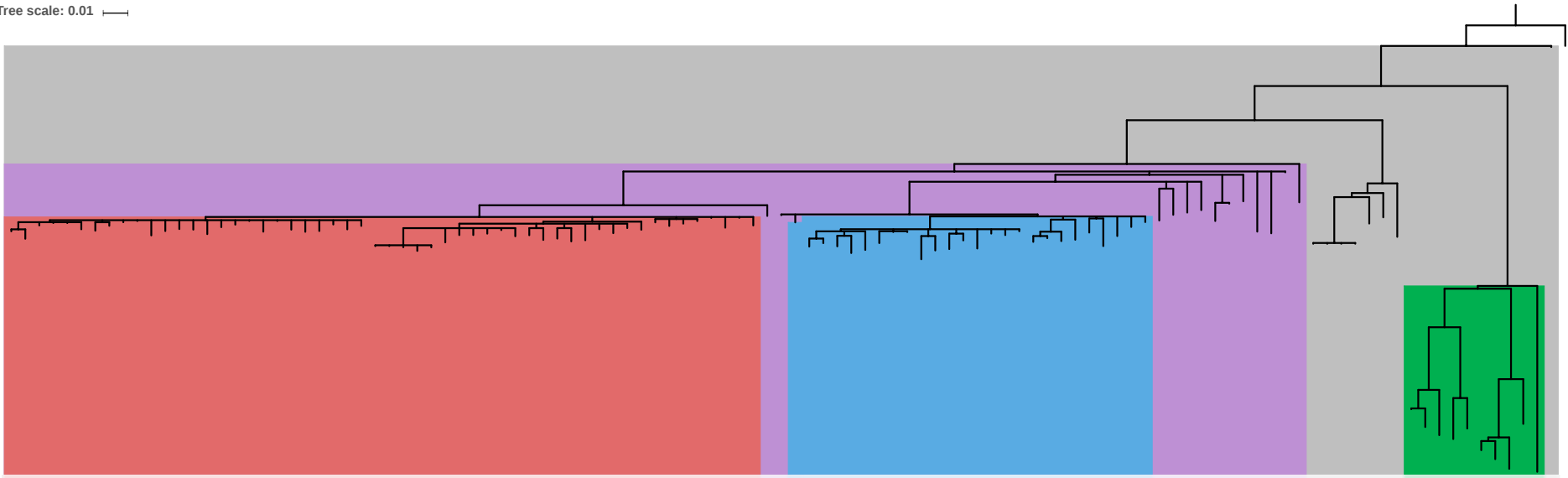

B

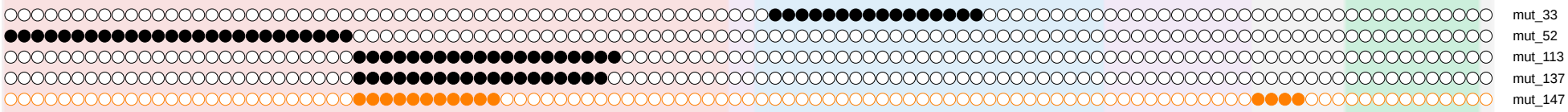

C

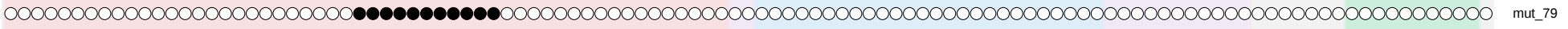

D

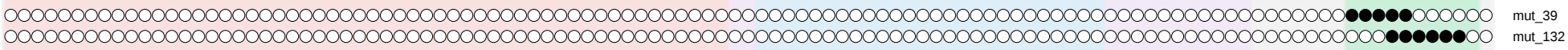

E

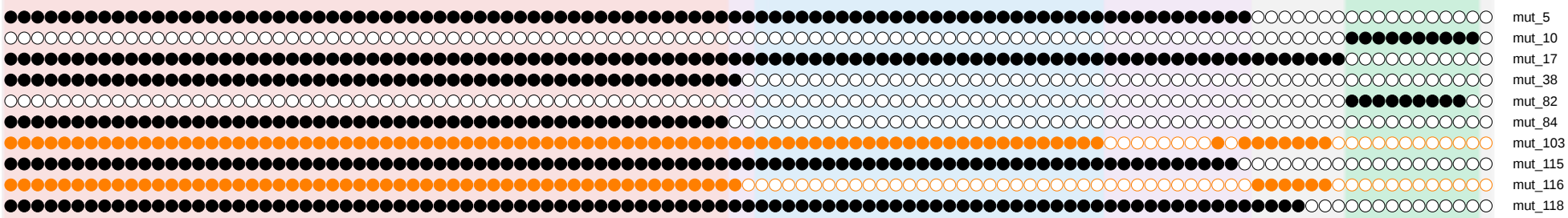

F

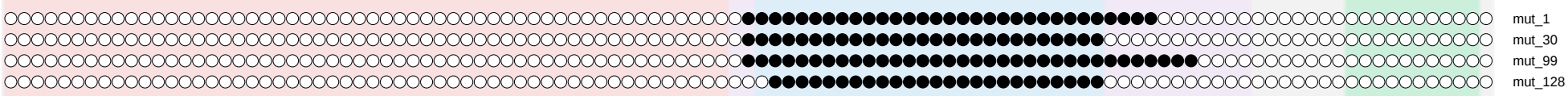

Supplement: S8 Fig — (A) Maximum Likelihood phylogeny of 111 B. multivorans isolates was elaborated using RaxML v. 7.0.4 with a GTR + gamma model and 1,000 bootstraps [95]. Here, we show all mutation profiles associated with antibiotic resistance prior to lineage control in black and with lineage control in orange. (B) resistance to both β-lactams, (C) to amikacin only, (D) to both aminoglycosides, (E) to both aminoglycosides and to ciprofloxacin, (F) and to ciprofloxacin only. A filled circle represents a SNP call in the corresponding isolate compared to the reference. (PDF) [file ppat.1007453.s008.pdf]

quinolone

ciprofloxacin

64

mut 147

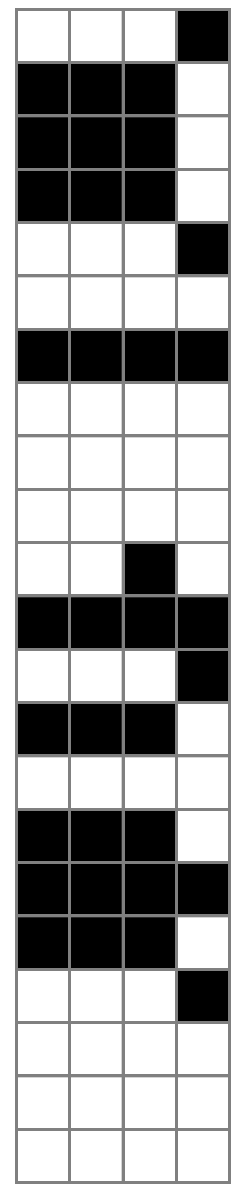

Supplement: S9 Fig — Mutational profiles were tested for association against six levels of antibiotic resistance (<16, <32, <64, <128, <256 and <512 MIC) to five antibiotics (amikacin, tobramycin, aztreonam, ceftazidime and ciprofloxacin). Black boxes show the levels of resistance at which the mutational profiles were statistically significant including multi-testing correction. Associations to ciprofloxacin antibiotic resistance are shown up to <128 MIC since no isolate had a MIC of 256 or greater in relation to that antibiotic. (PDF) [file ppat.1007453.s009.pdf]

**A**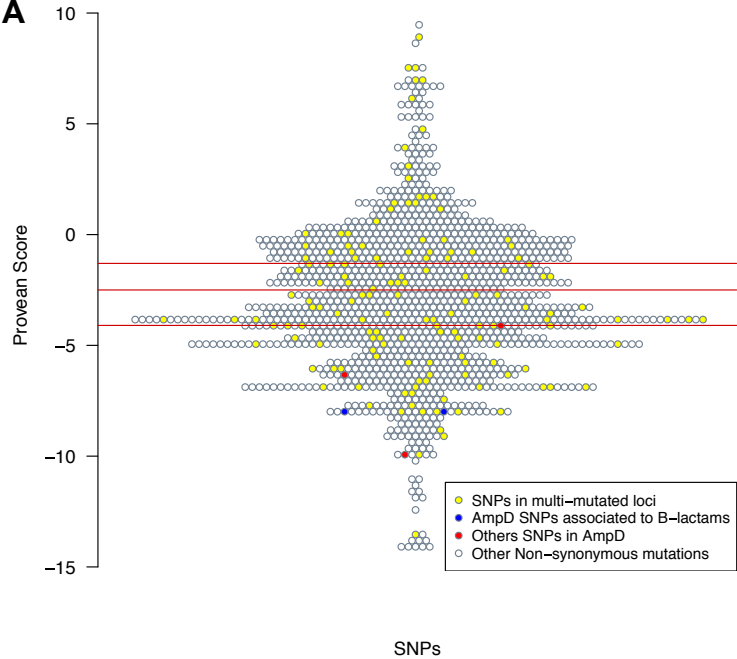**B**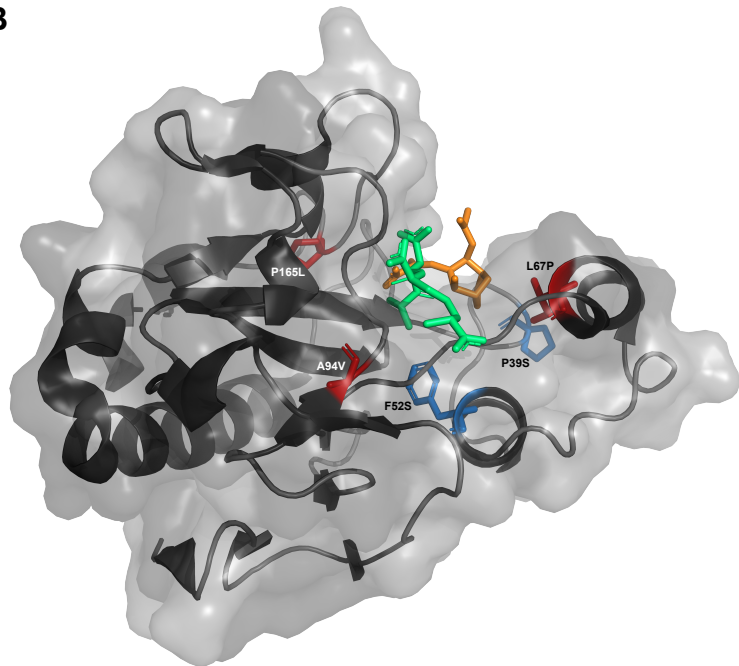

**B**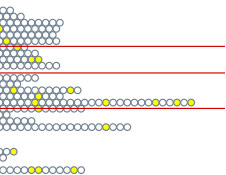

in multi-mutated loci  
SNPs associated to B-lactams  
SNPs in AmpD  
Non-synonymous mutations

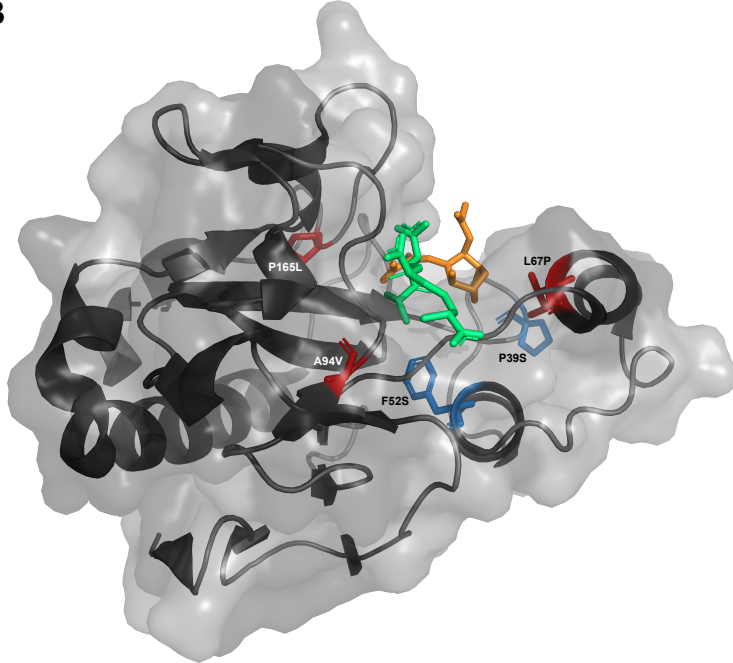

**A**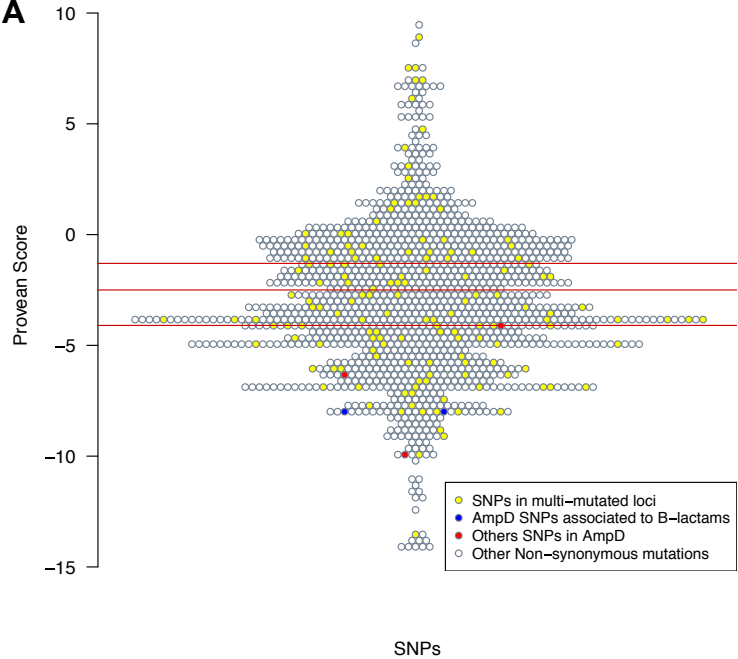**B**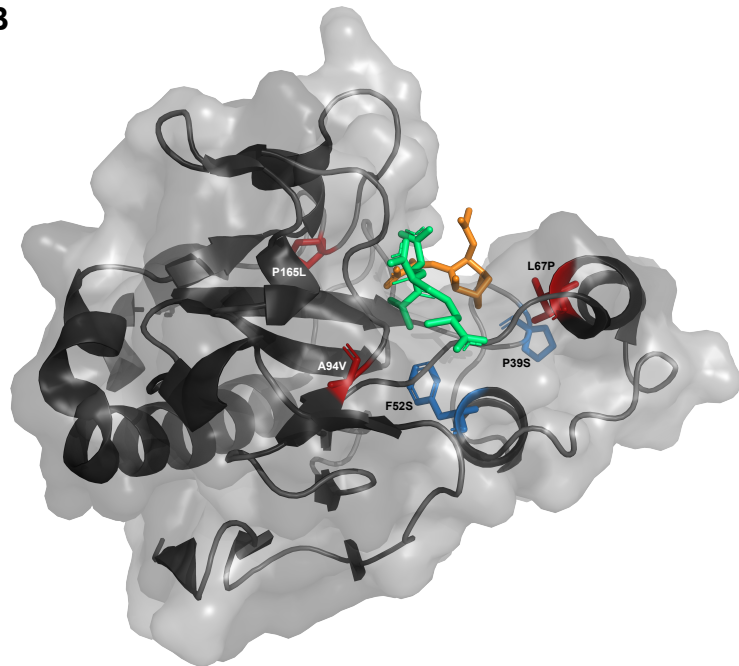

Supplement: S10 Fig — (A) Distribution of the PROVEAN scores of all identified non-synonymous substitutions highlighting SNPs in multi-mutated loci (yellow) and in the ampD gene (red or blue if associated to β -lactam resistance). Red lines represent thresholds from most specific (highest), to most sensitive (lowest) to determine if a mutation is deleterious to the function of the gene in which it occurs. (B) Crystal structure of protein product of AmpD (PDB ID:2Y2B, [96]) in complex with 1,6-anhydro-N-acetylmuramic acid and L-ala-gamma-D-glu-meso-diaminopimelic acid, which are associated to the cell-wall degradation pathway. Mutations found in our B. multivorans population are colored in red or blue (mutations associated with β-lactam resistance). (PDF) [file ppat.1007453.s010.pdf]

A

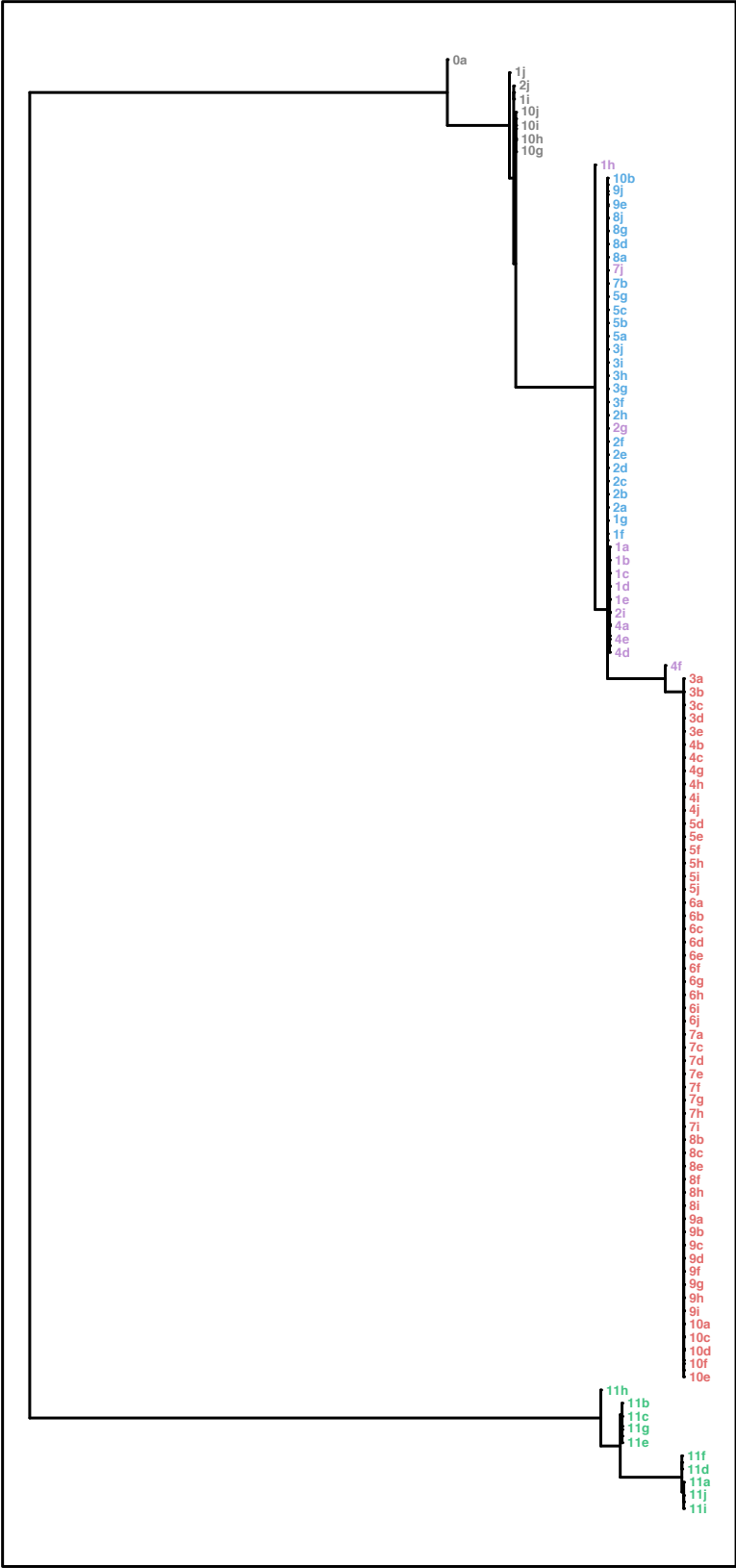

B

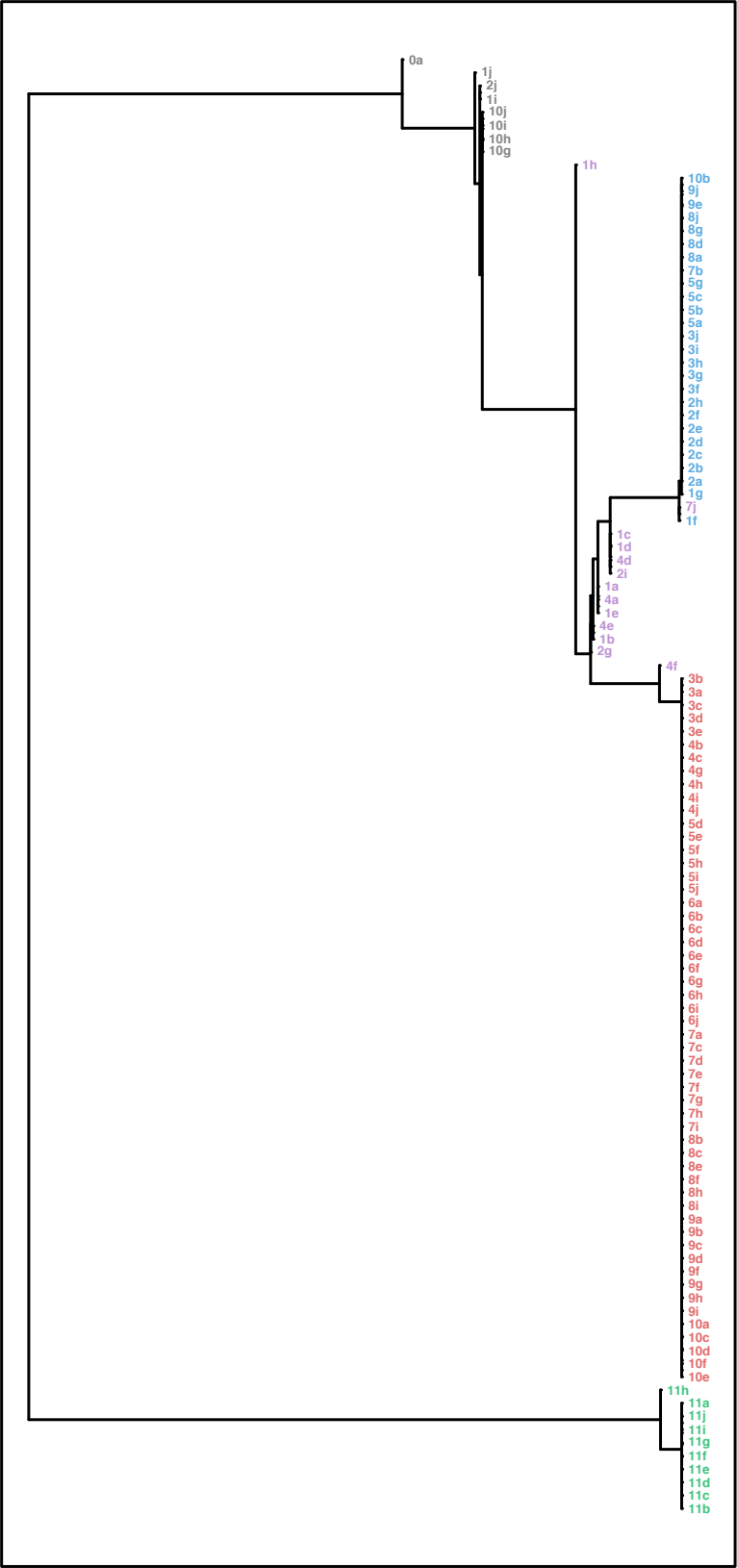

C

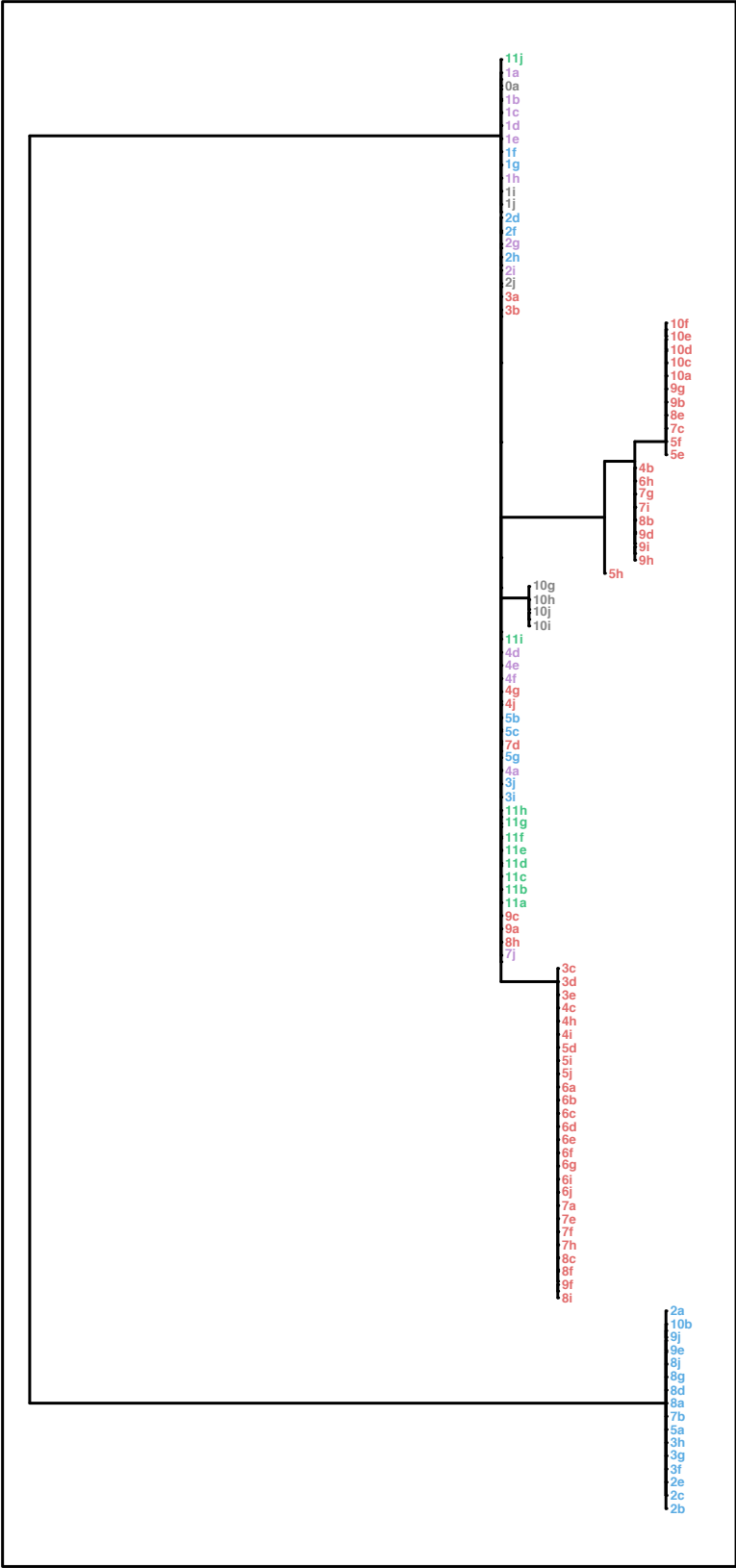

D

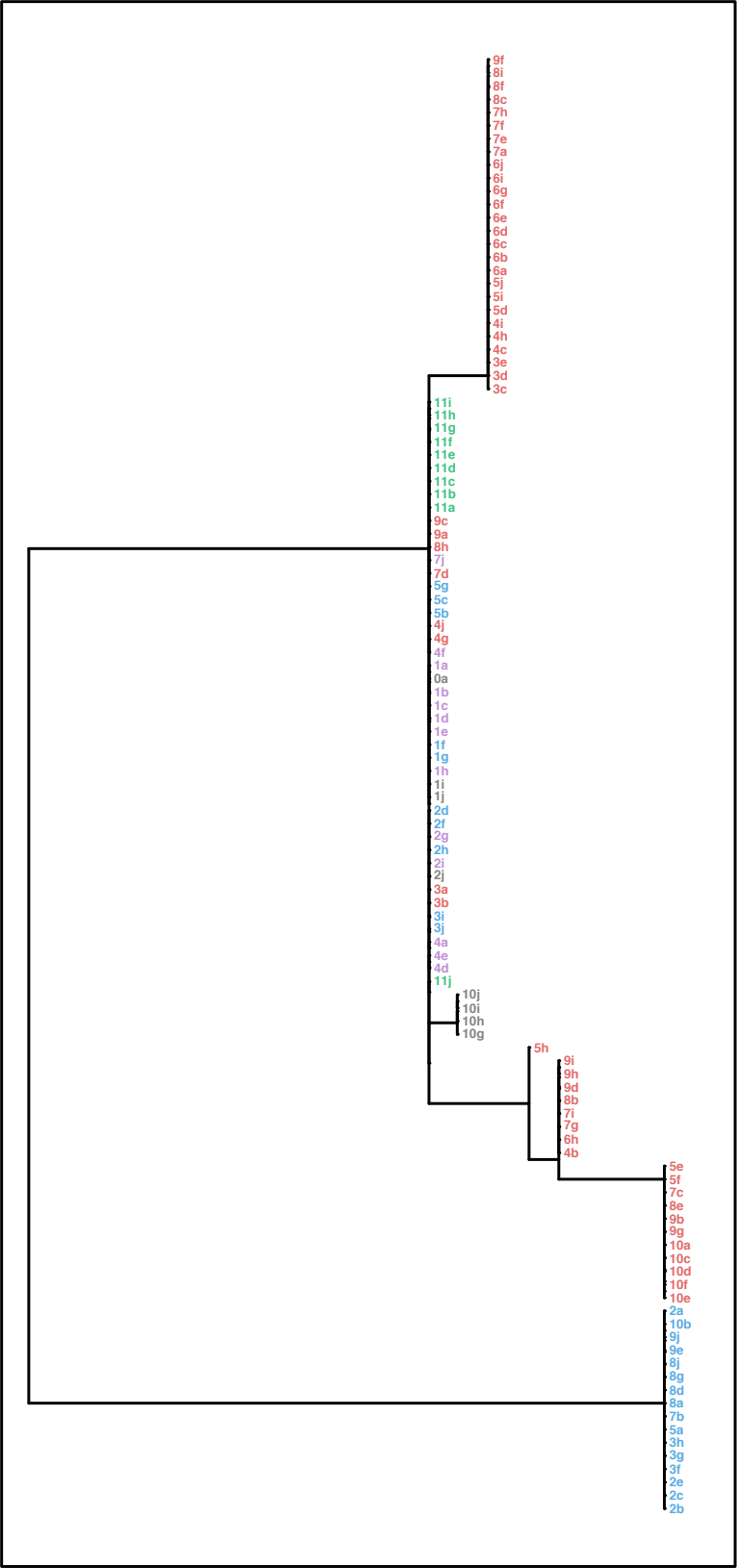

Supplement: S11 Fig — Maximum likelihood phylogenies for SNPs associated with resistance to A) Amikacin and Tobramycin, B) Ciprofloxacin, C) Aztreonam, and D) Ceftazidime were recreated in MEGA7 using the GTR model and 500 bootstrap iteration [81]. Each phylogeny was midpoint rooted. (PDF) [file ppat.1007453.s011.pdf]

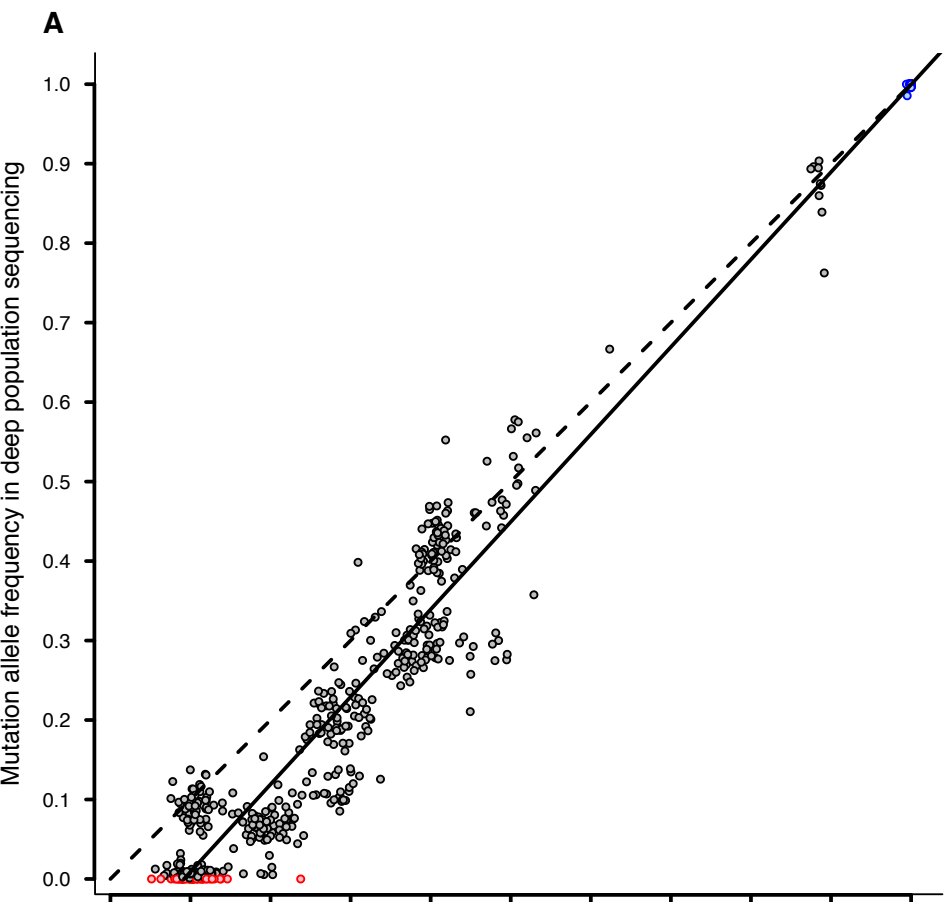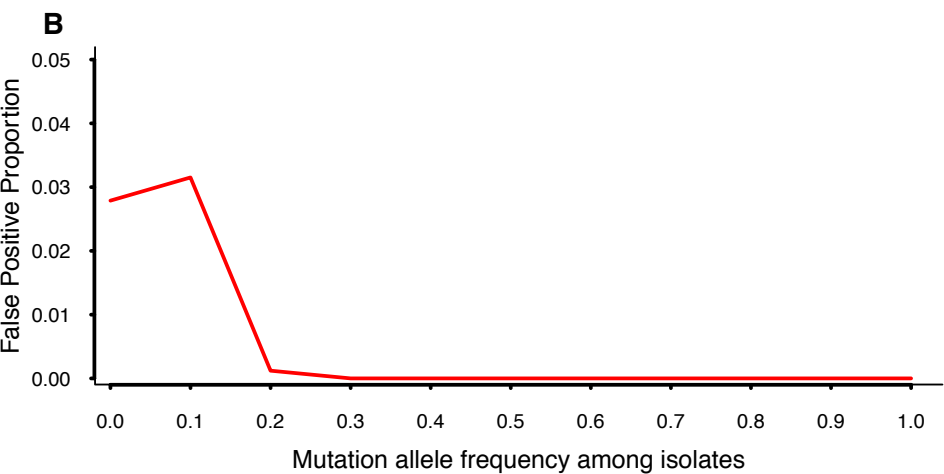

Supplement: S12 Fig — Sequencing reads from each isolate from the post-transplant sample were rarified to 1/10th of the number of reads in the population sequencing experiment; then they were combined so that the number of reads would be the same for both experiments. Sequencing reads from the population and single isolate experiments were mapped to the same reference as described above. Mutation allele frequencies for both experiments were calculated using the quality thresholds described by Lieberman et al. [53]. (A) Grey circles represent mutation allele frequencies in the deep population sequencing experiment (y axis) versus in single isolate sequencing (x axis). The dashed line represents the x = y function and the solid line is the best fit line taking into account all data points (R2 = 0.9928, 95% confidence interval = 0.9918–0.9937). Red circles represent alleles found in the single isolate sequencing experiment but not in the deep sequencing one. Fixed mutations between the reference and all the post-transplant isolates are colored blue. (B) Proportion of false positives in the single isolate sequencing experiment. (PDF) [file ppat.1007453.s012.pdf]

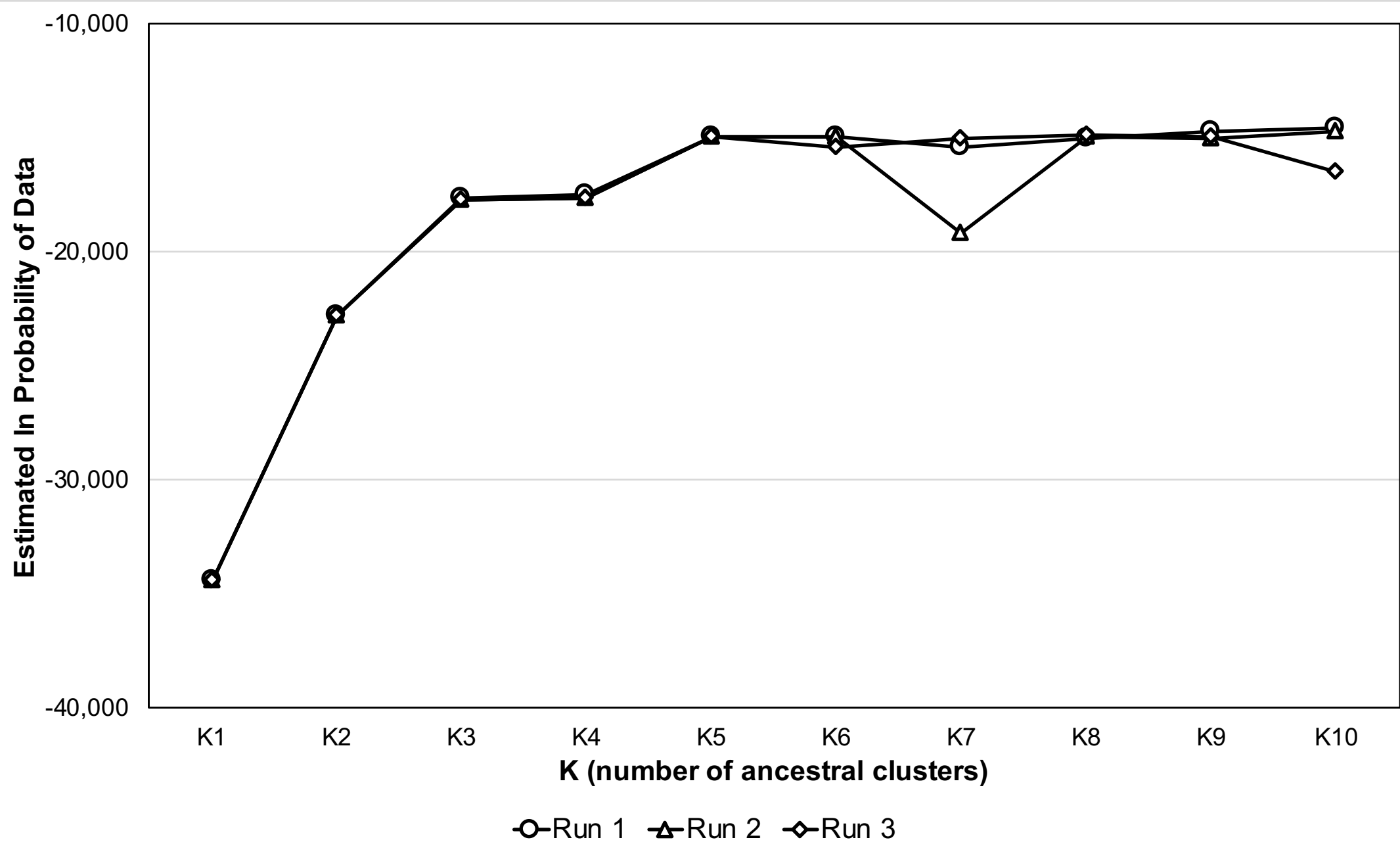

Supplement: S13 Fig — We ran three independent chains for each K between one and ten. The estimated ln probability of data plateaus at K = 3 in all chains. (PDF) [file ppat.1007453.s013.pdf]

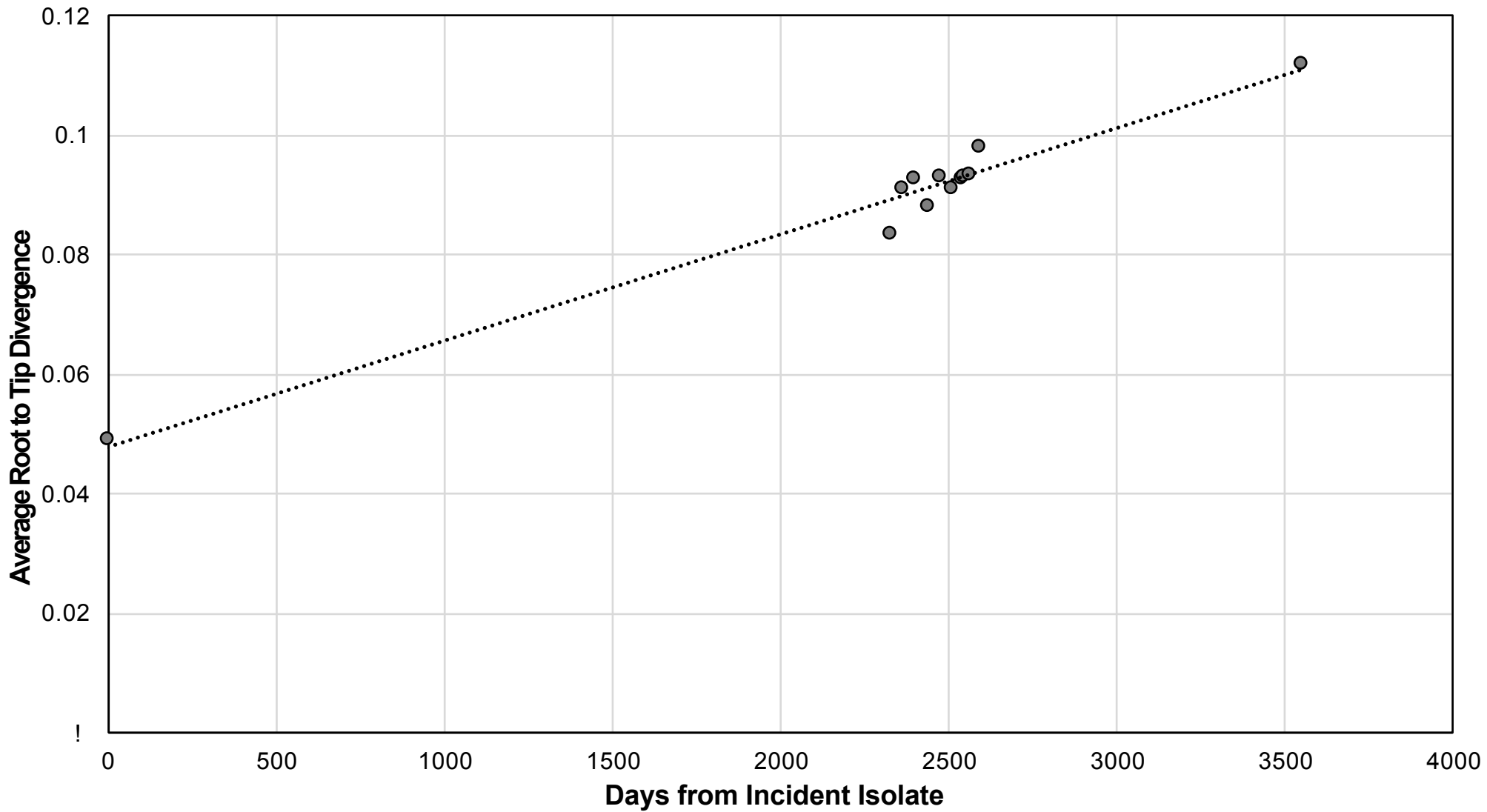

Supplement: S14 Fig — Each circle represents the average root-to-tip distance of the isolates from the respective sampling time point. The resulting trend shows that the inferred molecular clock was consistent with the changes seen in our isolates through time (R2 = 0.97, P < 0.0001). (PDF) [file ppat.1007453.s014.pdf]
