# Supplementary material for: A genome-wide association analysis reveals a potential role for recombination in the evolution of antimicrobial resistance in Burkholderia multivorans
Source: PLoS Pathog. 2018 Dec 7;14(12):e1007453. doi: 10.1371/journal.ppat.1007453 (PMC6300292; doi:10.1371/journal.ppat.1007453)
Supplement: S7 Fig — Here, “0”s and “1”s represent the reference or alternative base, respectively, at each SNP position for each strain. SNP1 is the only position where only Strain1 has a base alternative to the reference. Hence, mutational profile 1, 1-0-0-0, comprises only one SNP. On the other hand, Strain4 is the only strain with a variant base for positions SNP2 and SNP3. Therefore, mutational profile 2, 0-0-0-1, comprises SNP2 and SNP3. (PDF) [file ppat.1007453.s007.pdf]

	SNP1	SNP2	SNP3	SNP4	SNP5	SNP6	SNP7	SNP8
Strain1	1	0	0	0	1	1	1	1
Strain2	0	0	0	0	0	0	0	1
Strain3	0	0	0	1	0	0	0	0
Strain4	0	1	1	1	1	1	1	1
Mutational Profile	**1**	**2**		**3**		**4**		**5**
