# Supplementary material for: A genome-wide association analysis reveals a potential role for recombination in the evolution of antimicrobial resistance in Burkholderia multivorans
Source: PLoS Pathog. 2018 Dec 7;14(12):e1007453. doi: 10.1371/journal.ppat.1007453 (PMC6300292; doi:10.1371/journal.ppat.1007453)
Supplement: S1 Table — (DOCX) [file ppat.1007453.s016.docx]

| **Supplementary Table 1. Mutations Occurring in the Recombinogenic Region between RB & B Isolates** | | | |
| --- | --- | --- | --- |
| **Locus** | **Mutation** | **Type ^a^** | **Annotation** |
| BMUL_0250 | G56S ^b,c^ | NS | 50S ribosomal protein L4p (L1e) |
| BMUL_0250 | G56D ^b,c^ | NS | 50S ribosomal protein L4p (L1e) |
| BMUL_0254 | A44G | NS | 50S ribosomal protein L22 |
| BMUL_0258 | V5A | NS | 30S ribosomal protein S17 |
| BMUL_0258 | E54 | S | 30S ribosomal protein S17 |
| BMUL_0262 | E47G | NS | 30S ribosomal protein S14 |
| BMUL_0277 | D92G | NS | CutA1 divalent ion tolerance protein |
| BMUL_0278 | G299 | S | protein-disulfide reductase |
| BMUL_0278 | D579G | NS | protein-disulfide reductase |
| BMUL_0281 | N68 | S | cytochrome c class I |
| BMUL_0294 | A365T | NS | type IV pilus secretin PilQ |
| BMUL_0298 | A9V | NS | glycerol-3-phosphate transporter periplasmic binding protein |
| BMUL_0301 | M97T ^c^ | NS | glycerol-3-phosphate transporter ATP-binding subunit |
| BMUL_0301 | T277I ^c^ | NS | glycerol-3-phosphate transporter ATP-binding subunit |
| BMUL_0290 - _0291^d^ |  | IG | 1A family penicillin-binding protein^d^ & hypothetical protein ^e^ |
| BMUL_0303 - _0304 ^d^ |  | IG | OmpW family protein^d^ & hypothetical protein ^e^ |
| ^a^ NS, no-synonymous; S, synonymous; IG, intergenic  ^b^ Mutations occur in adjacent bases position in the same codon  ^c^ Mutation is associated with resistance to aminoglycosides and ciprofloxacin prior to population structure control  ^d^ Gene upstream of intergenic mutation  ^e^ Gene downstream of intergenic mutation | | | |
