# Supplementary material for: A genome-wide association analysis reveals a potential role for recombination in the evolution of antimicrobial resistance in Burkholderia multivorans
Source: PLoS Pathog. 2018 Dec 7;14(12):e1007453. doi: 10.1371/journal.ppat.1007453 (PMC6300292; doi:10.1371/journal.ppat.1007453)
Supplement: S2 Table — (DOCX) [file ppat.1007453.s017.docx]

| **Supplementary Table 2. Mutations Occurring in the Recombinogenic Regions among Post-Transplant isolates** | | | |
| --- | --- | --- | --- |
| **Locus** | **Mutation** | **Type ^a^** | **Annotation** |
| BMUL_2127 | A53T ^b,c^ | NS | ABC transporter-like protein |
| BMUL_2129 | T110A | NS | polar amino acid ABC transporter inner membrane subunit |
| BMUL_2133 | T116A | NS | hopanoid biosynthesis associated radical SAM protein HpnJ |
| BMUL_2133 | L99P | NS | hopanoid biosynthesis associated radical SAM protein HpnJ |
| BMUL_2138 | A225 | S | pyridoxal kinase |
| BMUL_2139 | L666P | NS | phospholipase C |
| BMUL_2146 | Y584H | NS | ABC transporter-like protein |
| BMUL_2149 | T44 | S | electron transport protein SCO1/SenC |
| BMUL_2152 | Q165* | NS | N-acetyltransferase GCN5 |
| BMUL_2153 | N128S | NS | pirin domain-containing protein |
| BMUL_2157 | I50T | NS | hypothetical protein |
| BMUL_2168 | Q149R | NS | DNA repair protein RecO |
| BMUL_2170 | A302V | NS | ribonuclease III |
| BMUL_2172 | D469G | NS | GTP-binding protein LepA |
| BMUL_2174 | S410L | NS | protease Do |
| BMUL_2177 | D134N | NS | RNA polymerase sigma factor RpoE |
| BMUL_2179 | A145G | NS | 3-oxoacyl-(acyl carrier protein) synthase II |
| BMUL_2179 |  | PR | 3-oxoacyl-(acyl carrier protein) synthase II |
| BMUL_2180 | A64T ^b,c^ | NS | acyl carrier protein |
| BMUL_2180 |  | PR | acyl carrier protein |
| BMUL_2182 | A168V ^b^ | NS | malonyl CoA-acyl carrier protein transacylase |
| BMUL_2182 | H101Y | NS | malonyl CoA-acyl carrier protein transacylase |
| BMUL_2183 | S179G | NS | 3-oxoacyl-ACP synthase |
| BMUL_2189 | V312G | NS | peptidase S49 |
| BMUL_2194 | I78T | NS | molybdenum cofactor biosynthesis protein A |
| BMUL_2195 | D146G | NS | molybdopterin-guanine dinucleotide biosynthesis protein MobA |
| BMUL_2196 | D338G | NS | molybdenum cofactor synthesis domain-containing protein |
| BMUL_2200 | D193G | NS | D-isomer specific 2-hydroxyacid dehydrogenase |
| BMUL_2203 |  | PR | nicotinate phosphoribosyltransferase |
| BMUL_2205 | Q92R | NS | CreA family protein |
| BMUL_2220 | Y18H | NS | hypothetical protein |
| BMUL_2221 | F32S | NS | dihydrofolate reductase |
| BMUL_2221 | F158S | NS | dihydrofolate reductase |
| BMUL_2225 | Q123R | NS | oligoribonuclease |
| BMUL_2226 | K224R | NS | Ste24 endopeptidase |
| BMUL_2227 | V70A | NS | ribosome-associated GTPase |
| BMUL_2230 | L65P | NS | NUDIX hydrolase |
| BMUL_2233 |  | PR | 16S rRNA processing protein RimM |
| BMUL_2240 | F312L^b,c^ | NS | D-amino acid dehydrogenase small subunit |
| BMUL_2241 | T146A | NS | electron transfer flavoprotein subunit alpha |
| BMUL_2245 | G215E ^b,c^ | NS | DL-methionine transporter ATP-binding subunit |
| BMUL_2248 | W327R | NS | lytic murein transglycosylase B |
| BMUL_2256 | D35G | NS | integration host factor subunit beta |
| BMUL_2257 | D464 | S | 30S ribosomal protein S1 |
| BMUL_2257 | F163 | S | 30S ribosomal protein S1 |
| BMUL_2164 - _2165 ^d^ |  | IG | Two component, sigma54 specific, Fis family transcriptional regulator ^d^ & beta-hexosaminidase ^e^ |
| BMUL_2257 _ 2258 d |  | IG | 30S ribosomal protein S1^d^ & citidylate kinase (cmk)^e^ |
| ^a^ NS, no-synonymous; S, synonymous; IG, intergenic; PR, putative regulator  ^b^ Mutation is associated with resistance to aminoglycosides prior to population structure control  ^c^ Mutation is associated with resistance to ciprofloxacin prior to population structure control  ^d^ Two loci flanking intergenic mutation | | | |
