# Supplementary material for: A genome-wide association analysis reveals a potential role for recombination in the evolution of antimicrobial resistance in Burkholderia multivorans
Source: PLoS Pathog. 2018 Dec 7;14(12):e1007453. doi: 10.1371/journal.ppat.1007453 (PMC6300292; doi:10.1371/journal.ppat.1007453)
Supplement: S3 Table — (DOCX) [file ppat.1007453.s018.docx]

**Supplementary Table 3. NCBI BioProject ID, BioSample IDs and Genbank Accession IDs for genomes of isolates mentioned in current study.**

| **Isolate ID** | **BioProject ID** | **BioSample ID** | **Genbank Accession** |
| --- | --- | --- | --- |
| 11j | PRJNA475602 | SAMN09396524 | QLAI00000000 |
| 11i | PRJNA475602 | SAMN09396491 | QLAJ00000000 |
| 11h | PRJNA475602 | SAMN09396458 | QLAK00000000 |
| 11g | PRJNA475602 | SAMN09396432 | QLAL00000000 |
| 11f | PRJNA475602 | SAMN09396396 | QLAM00000000 |
| 11e | PRJNA475602 | SAMN09396238 | QLAN00000000 |
| 11d | PRJNA475602 | SAMN09396175 | QLAO00000000 |
| 11c | PRJNA475602 | SAMN09396142 | QLAP00000000 |
| 11b | PRJNA475602 | SAMN09396125 | QLAQ00000000 |
| 11a | PRJNA475602 | SAMN09396047 | QLAR00000000 |
| 10j | PRJNA475602 | SAMN09395990 | QLAS00000000 |
| 10i | PRJNA475602 | SAMN09395910 | QLAT00000000 |
| 10h | PRJNA475602 | SAMN09395894 | QLAU00000000 |
| 10g | PRJNA475602 | SAMN09395779 | QLAV00000000 |
| 10f | PRJNA475602 | SAMN09395764 | QLAW00000000 |
| 10e | PRJNA475602 | SAMN09395727 | QLAX00000000 |
| 10d | PRJNA475602 | SAMN09395696 | QLAY00000000 |
| 10c | PRJNA475602 | SAMN09395639 | QLAZ00000000 |
| 10b | PRJNA475602 | SAMN09395628 | QLBA00000000 |
| 10a | PRJNA475602 | SAMN09395594 | QLBB00000000 |
| 9j | PRJNA475602 | SAMN09395576 | QLBC00000000 |
| 9i | PRJNA475602 | SAMN09395563 | QLBD00000000 |
| 9h | PRJNA475602 | SAMN09395562 | QLBE00000000 |
| 9g | PRJNA475602 | SAMN09395555 | QLBF00000000 |
| 9f | PRJNA475602 | SAMN09395547 | QLBG00000000 |
| 9e | PRJNA475602 | SAMN09395546 | QLBH00000000 |
| 9d | PRJNA475602 | SAMN09395545 | QLBI00000000 |
| 9c | PRJNA475602 | SAMN09395541 | QLBJ00000000 |
| 9b | PRJNA475602 | SAMN09395528 | QLBK00000000 |
| 9a | PRJNA475602 | SAMN09395527 | QLBL00000000 |
| 8j | PRJNA475602 | SAMN09395526 | QLBM00000000 |
| 8i | PRJNA475602 | SAMN09395525 | QLBN00000000 |
| 8h | PRJNA475602 | SAMN09395510 | QLBO00000000 |
| 8g | PRJNA475602 | SAMN09395509 | QLBP00000000 |
| 8f | PRJNA475602 | SAMN09395502 | QLBQ00000000 |
| 8e | PRJNA475602 | SAMN09395498 | QLBR00000000 |
| 8d | PRJNA475602 | SAMN09395454 | QLBS00000000 |
| 8c | PRJNA475602 | SAMN09395453 | QLBT00000000 |
| 8b | PRJNA475602 | SAMN09395452 | QLBU00000000 |
| 8a | PRJNA475602 | SAMN09395451 | QLBV00000000 |
| 7j | PRJNA475602 | SAMN09395437 | QLBW00000000 |
| 7i | PRJNA475602 | SAMN09395436 | QLBX00000000 |
| 7h | PRJNA475602 | SAMN09395429 | QLBY00000000 |
| 7g | PRJNA475602 | SAMN09395425 | QLBZ00000000 |
| 7f | PRJNA475602 | SAMN09395424 | QLCA00000000 |
| 7e | PRJNA475602 | SAMN09395409 | QLCB00000000 |
| 7d | PRJNA475602 | SAMN09395408 | QLCC00000000 |
| 7c | PRJNA475602 | SAMN09395403 | QLCD00000000 |
| 7b | PRJNA475602 | SAMN09395398 | QLCE00000000 |
| 7a | PRJNA475602 | SAMN09395312 | QLCF00000000 |
| 6j | PRJNA475602 | SAMN09395309 | QLCG00000000 |
| 6i | PRJNA475602 | SAMN09395290 | QLCH00000000 |
| 6h | PRJNA475602 | SAMN09395277 | QLCI00000000 |
| 6g | PRJNA475602 | SAMN09395269 | QLCJ00000000 |
| 6f | PRJNA475602 | SAMN09395235 | QLCK00000000 |
| 6e | PRJNA475602 | SAMN09395231 | QLCL00000000 |
| 6d | PRJNA475602 | SAMN09395229 | QLCM00000000 |
| 6c | PRJNA475602 | SAMN09395204 | QLCN00000000 |
| 6b | PRJNA475602 | SAMN09395202 | QLCO00000000 |
| 6a | PRJNA475602 | SAMN09395201 | QLCP00000000 |
| 5j | PRJNA475602 | SAMN09395183 | QLCQ00000000 |
| 5i | PRJNA475602 | SAMN09395182 | QLCR00000000 |
| 5h | PRJNA475602 | SAMN09395176 | QLCS00000000 |
| 5g | PRJNA475602 | SAMN09395159 | QLCT00000000 |
| 5f | PRJNA475602 | SAMN09394991 | QLCU00000000 |
| 5e | PRJNA475602 | SAMN09394889 | QLCV00000000 |
| 5d | PRJNA475602 | SAMN09394870 | QLCW00000000 |
| 5c | PRJNA475602 | SAMN09394839 | QLCX00000000 |
| 5b | PRJNA475602 | SAMN09394794 | QLCY00000000 |
| 5a | PRJNA475602 | SAMN09394793 | QLCZ00000000 |
| 4j | PRJNA475602 | SAMN09394785 | QLDA00000000 |
| 4i | PRJNA475602 | SAMN09394782 | QLDB00000000 |
| 4h | PRJNA475602 | SAMN09394781 | QLDC00000000 |
| 4g | PRJNA475602 | SAMN09394780 | QLDD00000000 |
| 4f | PRJNA475602 | SAMN09394764 | QLDE00000000 |
| 4e | PRJNA475602 | SAMN09394763 | QLDF00000000 |
| 4d | PRJNA475602 | SAMN09394762 | QLDG00000000 |
| 4c | PRJNA475602 | SAMN09394761 | QLDH00000000 |
| 4b | PRJNA475602 | SAMN09394735 | QLDI00000000 |
| 4a | PRJNA475602 | SAMN09394719 | QLDJ00000000 |
| 3j | PRJNA475602 | SAMN09394654 | QLDK00000000 |
| 3i | PRJNA475602 | SAMN09394627 | QLDL00000000 |
| 3h | PRJNA475602 | SAMN09394558 | QLDM00000000 |
| 3g | PRJNA475602 | SAMN09394544 | QLDN00000000 |
| 3f | PRJNA475602 | SAMN09394462 | QLDO00000000 |
| 3e | PRJNA475602 | SAMN09394461 | QLDP00000000 |
| 3d | PRJNA475602 | SAMN09394402 | QLDQ00000000 |
| 3c | PRJNA475602 | SAMN09394401 | QLDR00000000 |
| 3b | PRJNA475602 | SAMN09394398 | QLDS00000000 |
| 3a | PRJNA475602 | SAMN09394397 | QLDT00000000 |
| 2j | PRJNA475602 | SAMN09394391 | QLDU00000000 |
| 2i | PRJNA475602 | SAMN09394363 | QLDV00000000 |
| 2h | PRJNA475602 | SAMN09394362 | QLDW00000000 |
| 2g | PRJNA475602 | SAMN09394361 | QLDX00000000 |
| 2f | PRJNA475602 | SAMN09394360 | QLDY00000000 |
| 2e | PRJNA475602 | SAMN09394359 | QLDZ00000000 |
| 2d | PRJNA475602 | SAMN09394358 | QLEA00000000 |
| 2c | PRJNA475602 | SAMN09394357 | QLEB00000000 |
| 2b | PRJNA475602 | SAMN09394356 | QLEC00000000 |
| 2a | PRJNA475602 | SAMN09394355 | QLED00000000 |
| 1j | PRJNA475602 | SAMN09394354 | QLEE00000000 |
| 1i | PRJNA475602 | SAMN09394344 | QLEF00000000 |
| 1h | PRJNA475602 | SAMN09394343 | QLEG00000000 |
| 1g | PRJNA475602 | SAMN09394318 | QLEH00000000 |
| 1f | PRJNA475602 | SAMN09394303 | QLEI00000000 |
| 1e | PRJNA475602 | SAMN09394302 | QLEJ00000000 |
| 1d | PRJNA475602 | SAMN09394283 | QLEK00000000 |
| 1c | PRJNA475602 | SAMN09394274 | QLEL00000000 |
| 1b | PRJNA475602 | SAMN09394273 | QLEM00000000 |
| 1a | PRJNA475602 | SAMN09394272 | QLEN00000000 |
| 0a | PRJNA475602 | SAMN09396534 | QLEO00000000 |
