# Supplementary material for: A genome-wide association analysis reveals a potential role for recombination in the evolution of antimicrobial resistance in Burkholderia multivorans
Source: PLoS Pathog. 2018 Dec 7;14(12):e1007453. doi: 10.1371/journal.ppat.1007453 (PMC6300292; doi:10.1371/journal.ppat.1007453)
Supplement: S15 Fig — We recreated the phylogenies for each of the identified recombinogenic regions using the Maximum Likelihood method as implemented in MEGA7 with the GTR model and 500 bootstrap iterations [74]. The labels of each phylogeny correspond to the labels in Fig 2E. Each tree was rooted midpoint. (PDF) [file ppat.1007453.s015.pdf]

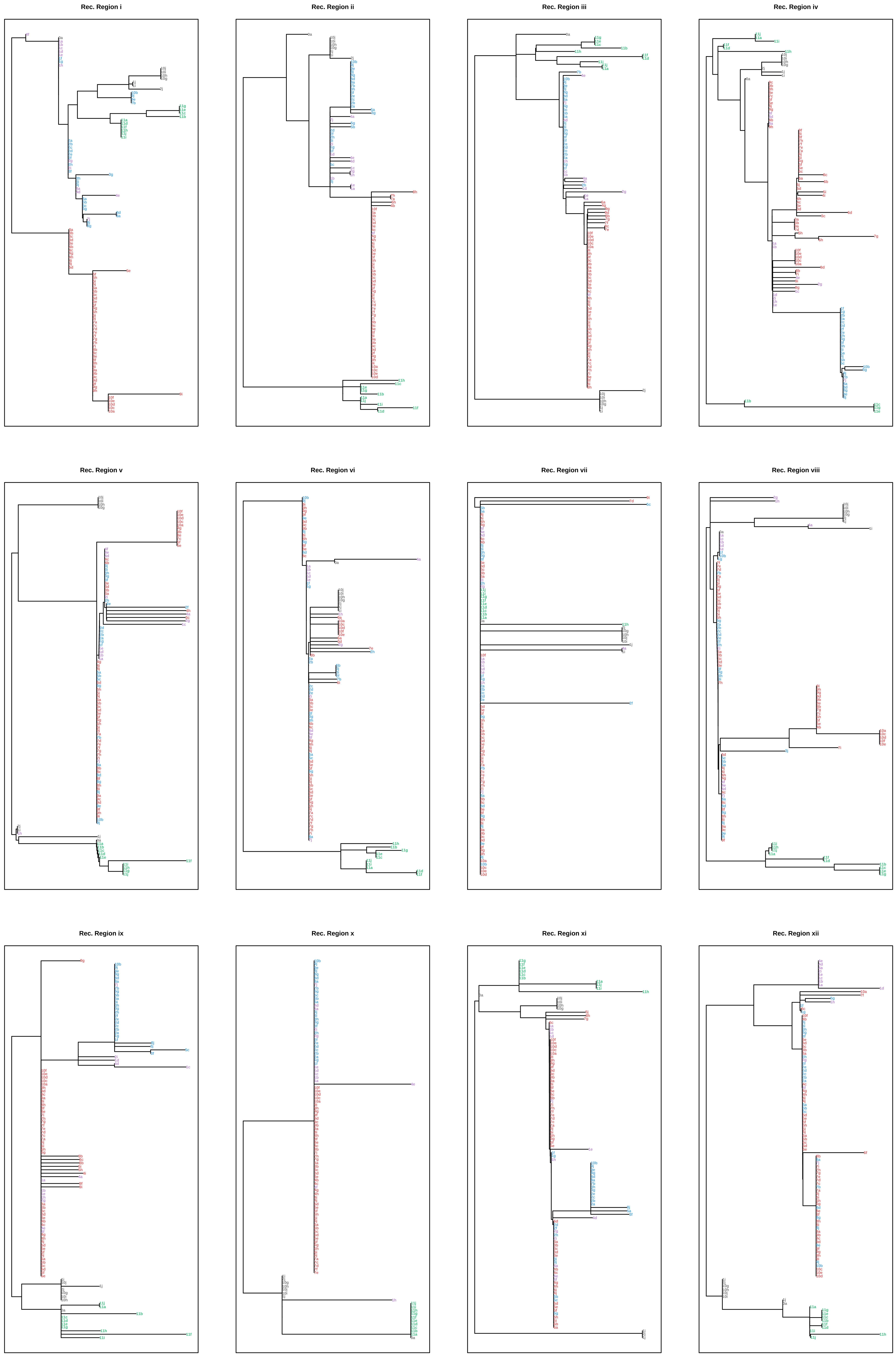
Rec. Region i
Rec. Region ii
Rec. Region iii
Rec. Region iv
Rec. Region v
Rec. Region vi
Rec. Region vii
Rec. Region viii
Rec. Region ix
Rec. Region x
Rec. Region xi
Rec. Region xii
